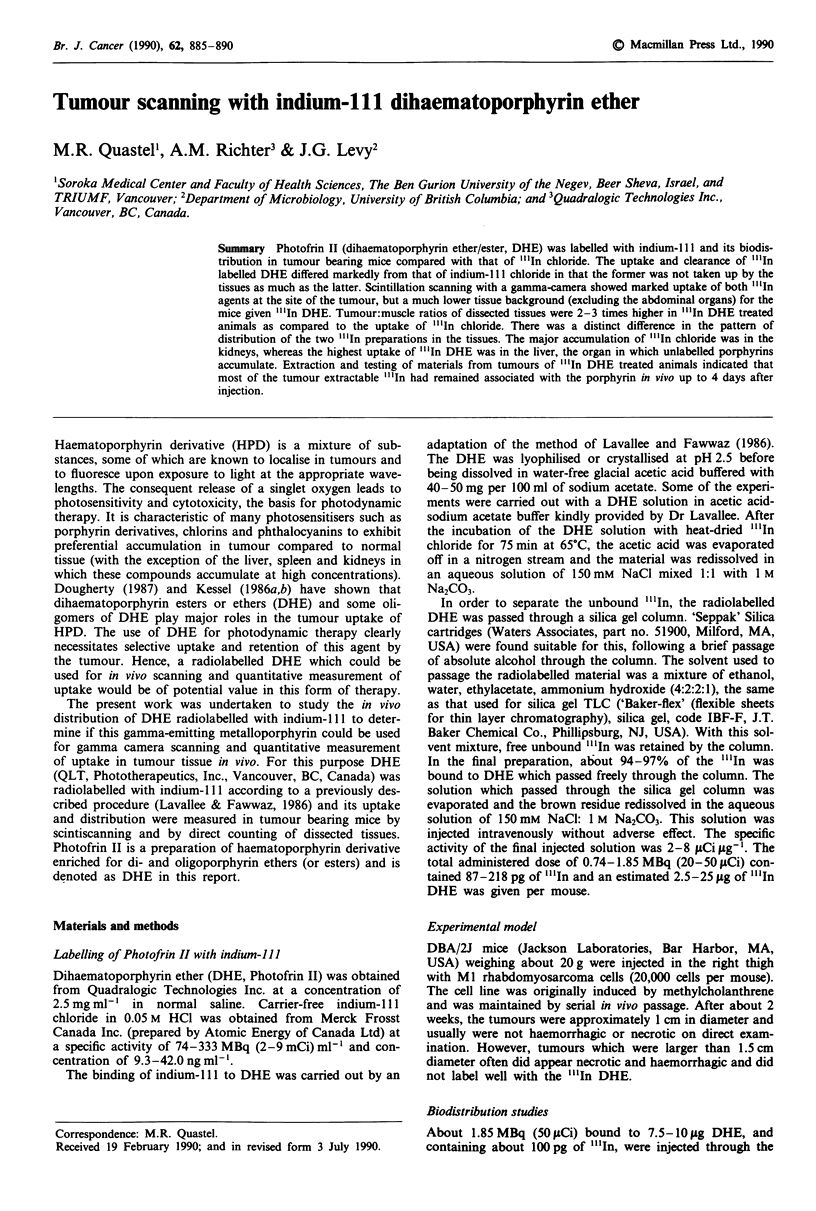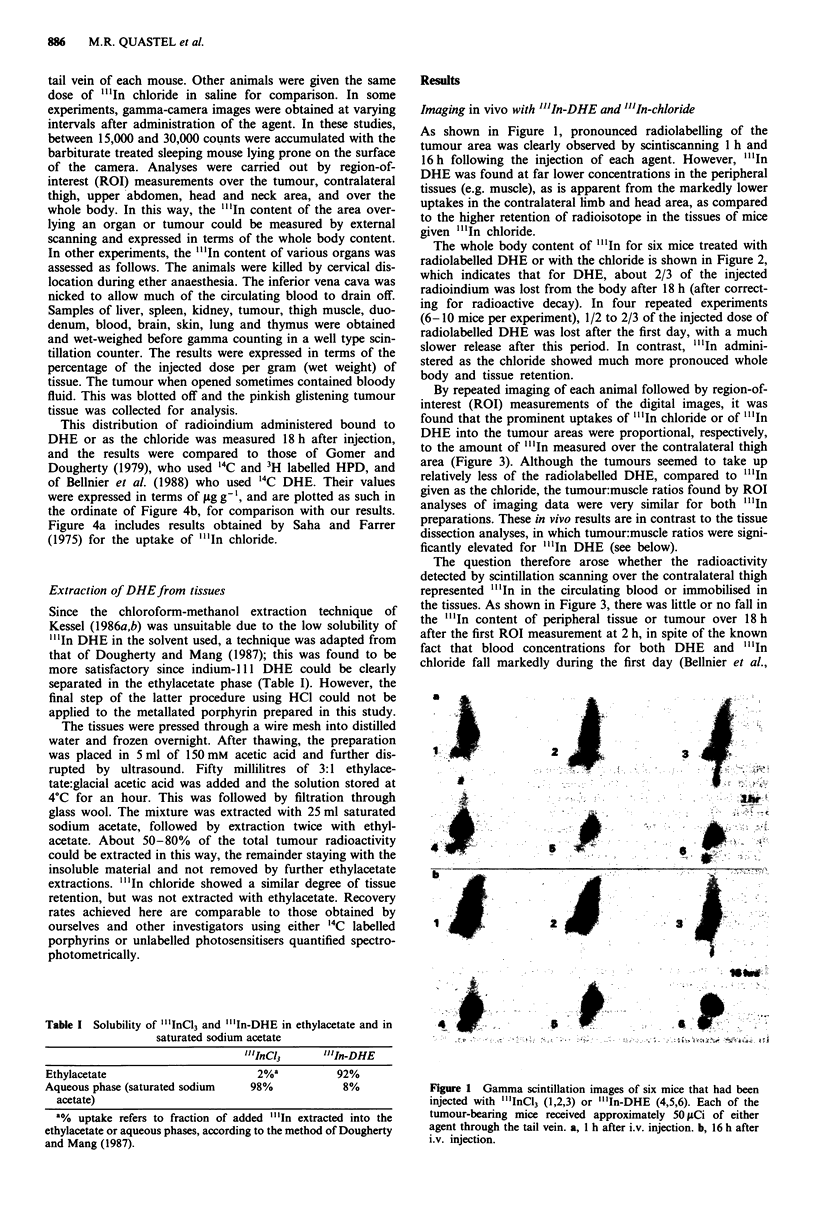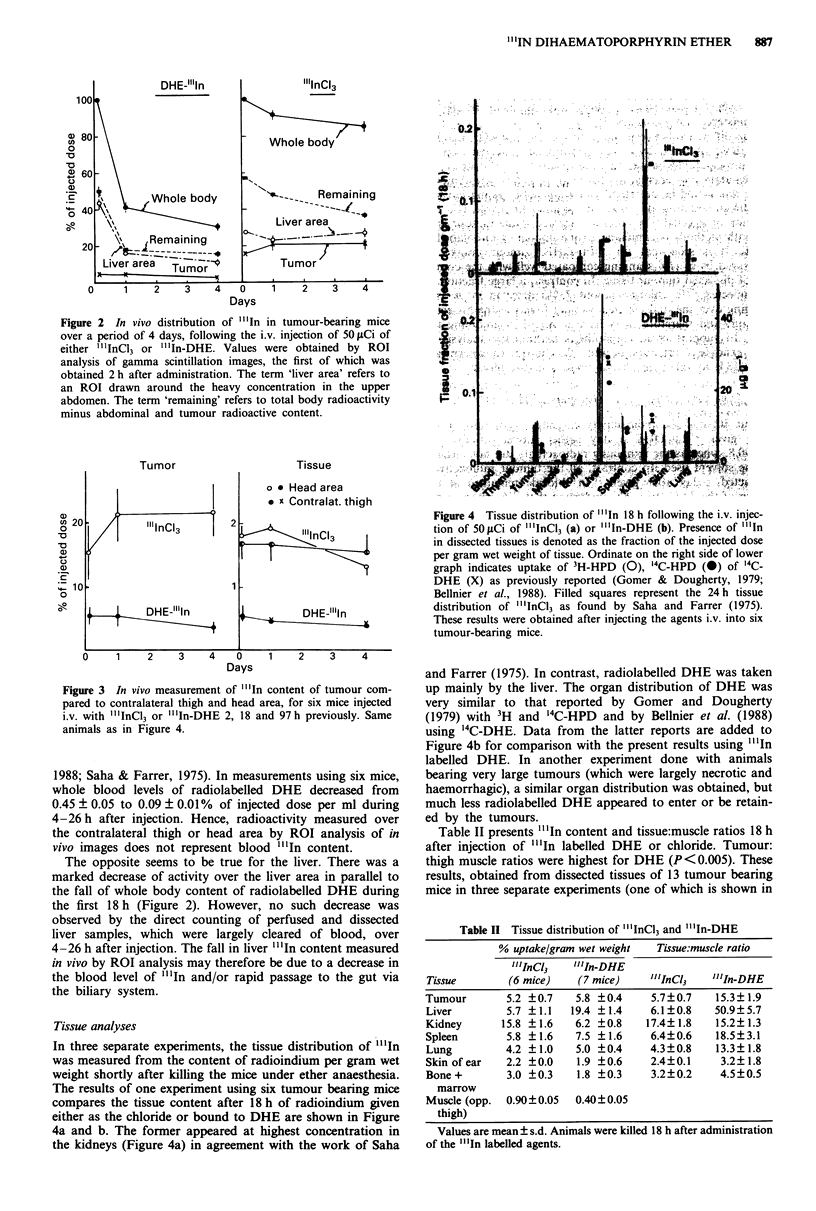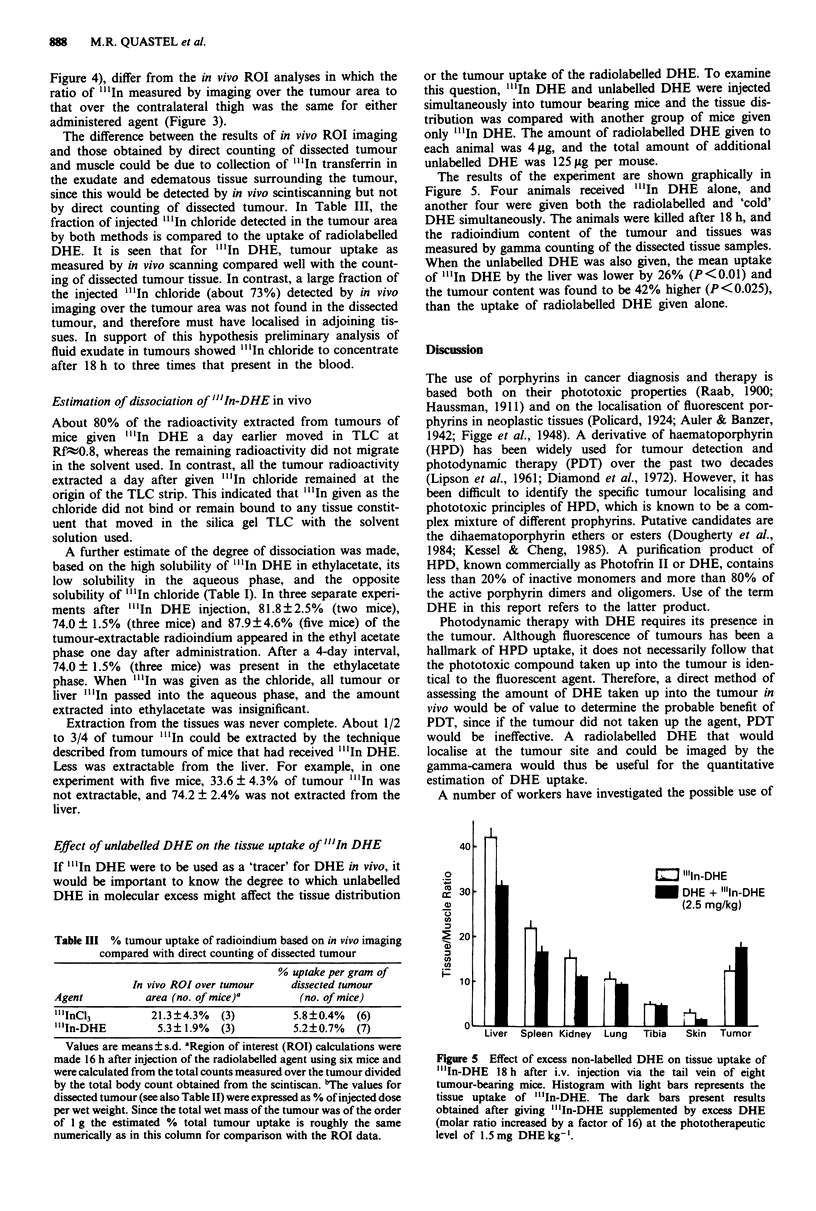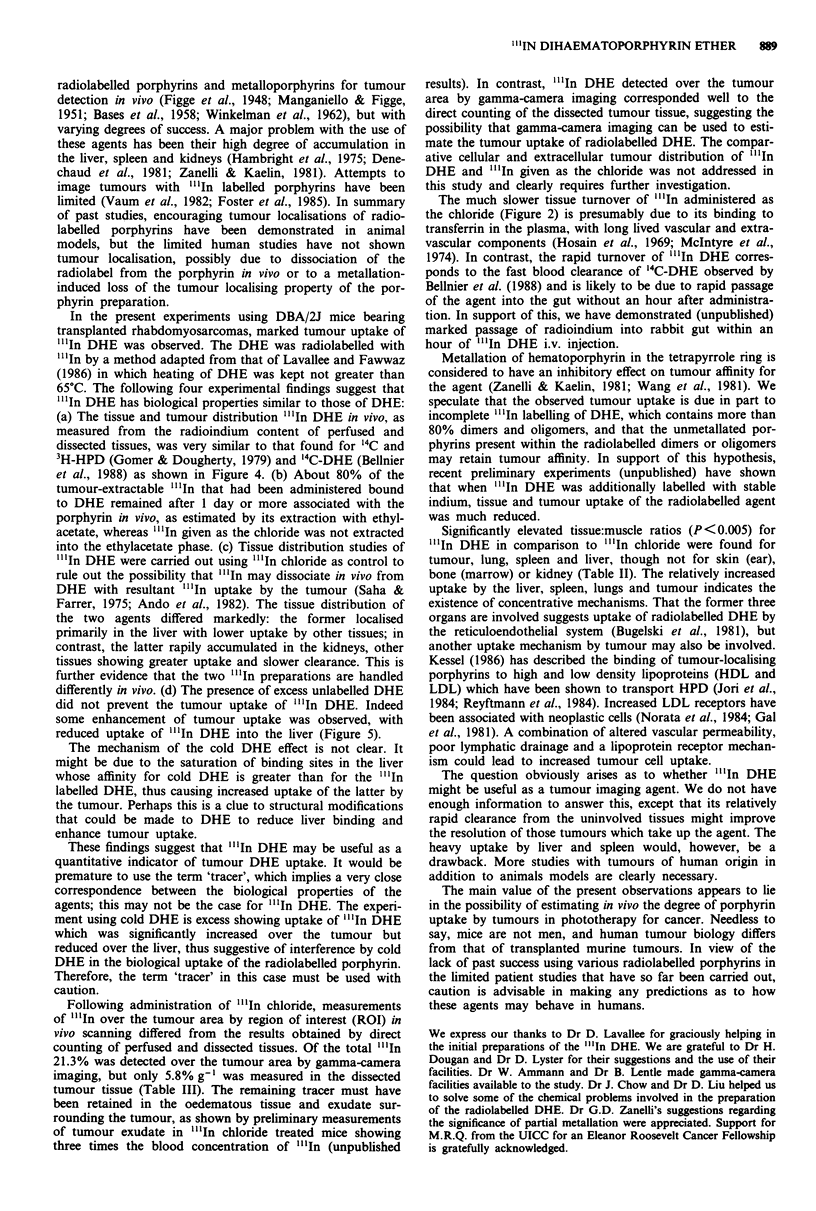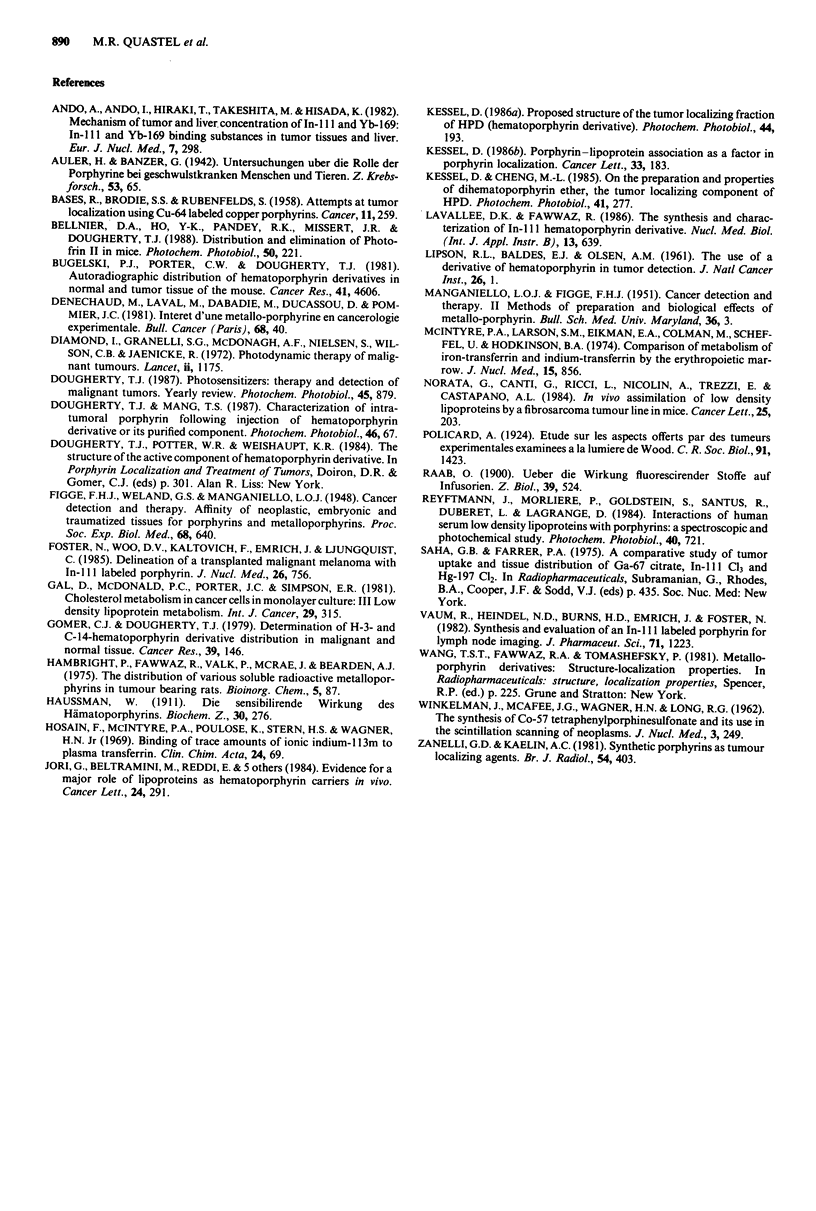# Tumour scanning with indium-111 dihaematoporphyrin ether.

**DOI:** 10.1038/bjc.1990.403

**Published:** 1990-12

**Authors:** M. R. Quastel, A. M. Richter, J. G. Levy

**Affiliations:** Soroka Medical Center, Ben Gurion University of the Negev, Beer Sheva, Israel.

## Abstract

**Images:**


Br. J. Cancer (1990), 62, 885–890 © Macmillan Press Ltd., 1990

### Tumour scanning with indium-111 dihaematoporphyrin ether

M.R. Quastel[1], A.M. Richter[3] & J.G. Levy[2]

[1]*Soroka Medical Center and Faculty of Health Sciences, The Ben Gurion University of the Negev, Beer Sheva, Israel, and TRIUMF, Vancouver;* [2]*Department of Microbiology, University of British Columbia; and* [3]*Quadralogic Technologies Inc., Vancouver, BC, Canada.*

**Summary** Photofrin II (dihaematoporphyrin ether/ester, DHE) was labelled with indium-111 and its biodistribution in tumour bearing mice compared with that of $^{111}$In chloride. The uptake and clearance of $^{111}$In labelled DHE differed markedly from that of indium-111 chloride in that the former was not taken up by the tissues as much as the latter. Scintillation scanning with a gamma-camera showed marked uptake of both $^{111}$In agents at the site of the tumour, but a much lower tissue background (excluding the abdominal organs) for the mice given $^{111}$In DHE. Tumour:muscle ratios of dissected tissues were 2–3 times higher in $^{111}$In DHE treated animals as compared to the uptake of $^{111}$In chloride. There was a distinct difference in the pattern of distribution of the two $^{111}$In preparations in the tissues. The major accumulation of $^{111}$In chloride was in the kidneys, whereas the highest uptake of $^{111}$In DHE was in the liver, the organ in which unlabelled porphyrins accumulate. Extraction and testing of materials from tumours of $^{111}$In DHE treated animals indicated that most of the tumour extractable $^{111}$In had remained associated with the porphyrin *in vivo* up to 4 days after injection.

Haematoporphyrin derivative (HPD) is a mixture of substances, some of which are known to localise in tumours and to fluoresce upon exposure to light at the appropriate wavelengths. The consequent release of a singlet oxygen leads to photosensitivity and cytotoxicity, the basis for photodynamic therapy. It is characteristic of many photosensitisers such as porphyrin derivatives, chlorins and phthalocyanins to exhibit preferential accumulation in tumour compared to normal tissue (with the exception of the liver, spleen and kidneys in which these compounds accumulate at high concentrations). Dougherty (1987) and Kessel (1986*a,b*) have shown that dihaematoporphyrin esters or ethers (DHE) and some oligomers of DHE play major roles in the tumour uptake of HPD. The use of DHE for photodynamic therapy clearly necessitates selective uptake and retention of this agent by the tumour. Hence, a radiolabelled DHE which could be used for *in vivo* scanning and quantitative measurement of uptake would be of potential value in this form of therapy.

The present work was undertaken to study the *in vivo* distribution of DHE radiolabelled with indium-111 to determine if this gamma-emitting metalloporphyrin could be used for gamma camera scanning and quantitative measurement of uptake in tumour tissue *in vivo*. For this purpose DHE (QLT, Phototherapeutics, Inc., Vancouver, BC, Canada) was radiolabelled with indium-111 according to a previously described procedure (Lavallee & Fawwaz, 1986) and its uptake and distribution were measured in tumour bearing mice by scintiscanning and by direct counting of dissected tissues. Photofrin II is a preparation of haematoporphyrin derivative enriched for di- and oligoporphyrin ethers (or esters) and is denoted as DHE in this report.

### Materials and methods

### *Labelling of Photofrin II with indium-111*

Dihaematoporphyrin ether (DHE, Photofrin II) was obtained from Quadralogic Technologies Inc. at a concentration of 2.5 mg ml$^{-1}$ in normal saline. Carrier-free indium-111 chloride in 0.05 M HCl was obtained from Merck Frosst Canada Inc. (prepared by Atomic Energy of Canada Ltd) at a specific activity of 74–333 MBq (2–9 mCi) ml$^{-1}$ and concentration of 9.3–42.0 ng ml$^{-1}$.

The binding of indium-111 to DHE was carried out by an adaptation of the method of Lavallee and Fawwaz (1986). The DHE was lyophilised or crystallised at pH 2.5 before being dissolved in water-free glacial acetic acid buffered with 40–50 mg per 100 ml of sodium acetate. Some of the experiments were carried out with a DHE solution in acetic acid-sodium acetate buffer kindly provided by Dr Lavallee. After the incubation of the DHE solution with heat-dried $^{111}$In chloride for 75 min at 65°C, the acetic acid was evaporated off in a nitrogen stream and the material was redissolved in an aqueous solution of 150 mM NaCl mixed 1:1 with 1 M $Na_2CO_3$.

In order to separate the unbound $^{111}$In, the radiolabelled DHE was passed through a silica gel column. 'Seppak' Silica cartridges (Waters Associates, part no. 51900, Milford, MA, USA) were found suitable for this, following a brief passage of absolute alcohol through the column. The solvent used to passage the radiolabelled material was a mixture of ethanol, water, ethylacetate, ammonium hydroxide (4:2:2:1), the same as that used for silica gel TLC ('Baker-flex' (flexible sheets for thin layer chromatography), silica gel, code IBF-F, J.T. Baker Chemical Co., Phillipsburg, NJ, USA). With this solvent mixture, free unbound $^{111}$In was retained by the column. In the final preparation, about 94–97% of the $^{111}$In was bound to DHE which passed freely through the column. The solution which passed through the silica gel column was evaporated and the brown residue redissolved in the aqueous solution of 150 mM NaCl: 1 M $Na_2CO_3$. This solution was injected intravenously without adverse effect. The specific activity of the final injected solution was 2–8 $\mu$Ci $\mu$g$^{-1}$. The total administered dose of 0.74–1.85 MBq (20–50 $\mu$Ci) contained 87–218 pg of $^{111}$In and an estimated 2.5–25 $\mu$g of $^{111}$In DHE was given per mouse.

### *Experimental model*

DBA/2J mice (Jackson Laboratories, Bar Harbor, MA, USA) weighing about 20 g were injected in the right thigh with M1 rhabdomyosarcoma cells (20,000 cells per mouse). The cell line was originally induced by methylcholanthrene and was maintained by serial *in vivo* passage. After about 2 weeks, the tumours were approximately 1 cm in diameter and usually were not haemorrhagic or necrotic on direct examination. However, tumours which were larger than 1.5 cm diameter often did appear necrotic and haemorrhagic and did not label well with the $^{111}$In DHE.

### *Biodistribution studies*

About 1.85 MBq (50 $\mu$Ci) bound to 7.5–10 $\mu$g DHE, and containing about 100 pg of $^{111}$In, were injected through the

Correspondence: M.R. Quastel.
Received 19 February 1990; and in revised form 3 July 1990.

tail vein of each mouse. Other animals were given the same dose of $^{111}In$ chloride in saline for comparison. In some experiments, gamma-camera images were obtained at varying intervals after administration of the agent. In these studies, between 15,000 and 30,000 counts were accumulated with the barbiturate treated sleeping mouse lying prone on the surface of the camera. Analyses were carried out by region-of-interest (ROI) measurements over the tumour, contralateral thigh, upper abdomen, head and neck area, and over the whole body. In this way, the $^{111}In$ content of the area overlying an organ or tumour could be measured by external scanning and expressed in terms of the whole body content. In other experiments, the $^{111}In$ content of various organs was assessed as follows. The animals were killed by cervical dislocation during ether anaesthesia. The inferior vena cava was nicked to allow much of the circulating blood to drain off. Samples of liver, spleen, kidney, tumour, thigh muscle, duodenum, blood, brain, skin, lung and thymus were obtained and wet-weighed before gamma counting in a well type scintillation counter. The results were expressed in terms of the percentage of the injected dose per gram (wet weight) of tissue. The tumour when opened sometimes contained bloody fluid. This was blotted off and the pinkish glistening tumour tissue was collected for analysis.

This distribution of radioindium administered bound to DHE or as the chloride was measured 18 h after injection, and the results were compared to those of Gomer and Dougherty (1979), who used $^{14}C$ and $^{3}H$ labelled HPD, and of Bellnier *et al.* (1988) who used $^{14}C$ DHE. Their values were expressed in terms of $\mu g\ g^{-1}$, and are plotted as such in the ordinate of Figure 4b, for comparison with our results. Figure 4a includes results obtained by Saha and Farrer (1975) for the uptake of $^{111}In$ chloride.

### *Extraction of DHE from tissues*

Since the chloroform-methanol extraction technique of Kessel (1986*a,b*) was unsuitable due to the low solubility of $^{111}In$ DHE in the solvent used, a technique was adapted from that of Dougherty and Mang (1987); this was found to be more satisfactory since indium-111 DHE could be clearly separated in the ethylacetate phase (Table I). However, the final step of the latter procedure using HCl could not be applied to the metallated porphyrin prepared in this study.

The tissues were pressed through a wire mesh into distilled water and frozen overnight. After thawing, the preparation was placed in 5 ml of 150 mM acetic acid and further disrupted by ultrasound. Fifty millilitres of 3:1 ethylacetate:glacial acetic acid was added and the solution stored at 4°C for an hour. This was followed by filtration through glass wool. The mixture was extracted with 25 ml saturated sodium acetate, followed by extraction twice with ethylacetate. About 50–80% of the total tumour radioactivity could be extracted in this way, the remainder staying with the insoluble material and not removed by further ethylacetate extractions. $^{111}In$ chloride showed a similar degree of tissue retention, but was not extracted with ethylacetate. Recovery rates achieved here are comparable to those obtained by ourselves and other investigators using either $^{14}C$ labelled porphyrins or unlabelled photosensitisers quantified spectrophotometrically.

**Table I** Solubility of $^{111}InCl_3$ and $^{111}In$-DHE in ethylacetate and in saturated sodium acetate

| | $^{111}InCl_3$ | $^{111}In$-DHE |
|---|---|---|
| Ethylacetate | 2%[a] | 92% |
| Aqueous phase (saturated sodium acetate) | 98% | 8% |

[a]% uptake refers to fraction of added $^{111}In$ extracted into the ethylacetate or aqueous phases, according to the method of Dougherty and Mang (1987).

### Results

### *Imaging* in vivo *with $^{111}In$-DHE and $^{111}In$-chloride*

As shown in Figure 1, pronounced radiolabelling of the tumour area was clearly observed by scintiscanning 1 h and 16 h following the injection of each agent. However, $^{111}In$ DHE was found at far lower concentrations in the peripheral tissues (e.g. muscle), as is apparent from the markedly lower uptakes in the contralateral limb and head area, as compared to the higher retention of radioisotope in the tissues of mice given $^{111}In$ chloride.

The whole body content of $^{111}In$ for six mice treated with radiolabelled DHE or with the chloride is shown in Figure 2, which indicates that for DHE, about 2/3 of the injected radioindium was lost from the body after 18 h (after correcting for radioactive decay). In four repeated experiments (6–10 mice per experiment), 1/2 to 2/3 of the injected dose of radiolabelled DHE was lost after the first day, with a much slower release after this period. In contrast, $^{111}In$ administered as the chloride showed much more pronouced whole body and tissue retention.

By repeated imaging of each animal followed by region-of-interest (ROI) measurements of the digital images, it was found that the prominent uptakes of $^{111}In$ chloride or of $^{111}In$ DHE into the tumour areas were proportional, respectively, to the amount of $^{111}In$ measured over the contralateral thigh area (Figure 3). Although the tumours seemed to take up relatively less of the radiolabelled DHE, compared to $^{111}In$ given as the chloride, the tumour:muscle ratios found by ROI analyses of imaging data were very similar for both $^{111}In$ preparations. These *in vivo* results are in contrast to the tissue dissection analyses, in which tumour:muscle ratios were significantly elevated for $^{111}In$ DHE (see below).

The question therefore arose whether the radioactivity detected by scintillation scanning over the contralateral thigh represented $^{111}In$ in the circulating blood or immobilised in the tissues. As shown in Figure 3, there was little or no fall in the $^{111}In$ content of peripheral tissue or tumour over 18 h after the first ROI measurement at 2 h, in spite of the known fact that blood concentrations for both DHE and $^{111}In$ chloride fall markedly during the first day (Bellnier *et al.*,

**Figure 1** Gamma scintillation images of six mice that had been injected with $^{111}InCl_3$ (1,2,3) or $^{111}In$-DHE (4,5,6). Each of the tumour-bearing mice received approximately 50 $\mu$Ci of either agent through the tail vein. **a**, 1 h after i.v. injection. **b**, 16 h after i.v. injection.

**Figure 2** *In vivo* distribution of $^{111}$In in tumour-bearing mice over a period of 4 days, following the i.v. injection of 50 μCi of either $^{111}InCl_3$ or $^{111}$In-DHE. Values were obtained by ROI analysis of gamma scintillation images, the first of which was obtained 2 h after administration. The term 'liver area' refers to an ROI drawn around the heavy concentration in the upper abdomen. The term 'remaining' refers to total body radioactivity minus abdominal and tumour radioactive content.

**Figure 3** *In vivo* measurement of $^{111}$In content of tumour compared to contralateral thigh and head area, for six mice injected i.v. with $^{111}InCl_3$ or $^{111}$In-DHE 2, 18 and 97 h previously. Same animals as in Figure 4.

1988; Saha & Farrer, 1975). In measurements using six mice, whole blood levels of radiolabelled DHE decreased from 0.45 ± 0.05 to 0.09 ± 0.01% of injected dose per ml during 4–26 h after injection. Hence, radioactivity measured over the contralateral thigh or head area by ROI analysis of *in vivo* images does not represent blood $^{111}$In content.

The opposite seems to be true for the liver. There was a marked decrease of activity over the liver area in parallel to the fall of whole body content of radiolabelled DHE during the first 18 h (Figure 2). However, no such decrease was observed by the direct counting of perfused and dissected liver samples, which were largely cleared of blood, over 4–26 h after injection. The fall in liver $^{111}$In content measured *in vivo* by ROI analysis may therefore be due to a decrease in the blood level of $^{111}$In and/or rapid passage to the gut via the biliary system.

### Tissue analyses

In three separate experiments, the tissue distribution of $^{111}$In was measured from the content of radioindium per gram wet weight shortly after killing the mice under ether anaesthesia. The results of one experiment using six tumour bearing mice compares the tissue content after 18 h of radioindium given either as the chloride or bound to DHE are shown in Figure 4a and b. The former appeared at highest concentration in the kidneys (Figure 4a) in agreement with the work of Saha

**Figure 4** Tissue distribution of $^{111}$In 18 h following the i.v. injection of 50 μCi of $^{111}InCl_3$ (**a**) or $^{111}$In-DHE (**b**). Presence of $^{111}$In in dissected tissues is denoted as the fraction of the injected dose per gram wet weight of tissue. Ordinate on the right side of lower graph indicates uptake of $^{3}$H-HPD (O), $^{14}$C-HPD (●) of $^{14}$C-DHE (X) as previously reported (Gomer & Dougherty, 1979; Bellnier *et al.*, 1988). Filled squares represent the 24 h tissue distribution of $^{111}InCl_3$ as found by Saha and Farrer (1975). These results were obtained after injecting the agents i.v. into six tumour-bearing mice.

and Farrer (1975). In contrast, radiolabelled DHE was taken up mainly by the liver. The organ distribution of DHE was very similar to that reported by Gomer and Dougherty (1979) with $^{3}$H and $^{14}$C-HPD and by Bellnier *et al.* (1988) using $^{14}$C-DHE. Data from the latter reports are added to Figure 4b for comparison with the present results using $^{111}$In labelled DHE. In another experiment done with animals bearing very large tumours (which were largely necrotic and haemorrhagic), a similar organ distribution was obtained, but much less radiolabelled DHE appeared to enter or be retained by the tumours.

Table II presents $^{111}$In content and tissue:muscle ratios 18 h after injection of $^{111}$In labelled DHE or chloride. Tumour: thigh muscle ratios were highest for DHE ($P < 0.005$). These results, obtained from dissected tissues of 13 tumour bearing mice in three separate experiments (one of which is shown in

**Table II** Tissue distribution of $^{111}InCl_3$ and $^{111}$In-DHE

| | % uptake/gram wet weight | | Tissue:muscle ratio | |
|---|---|---|---|---|
| Tissue | $^{111}InCl_3$ (6 mice) | $^{111}$In-DHE (7 mice) | $^{111}InCl_3$ | $^{111}$In-DHE |
| Tumour | 5.2 ±0.7 | 5.8 ±0.4 | 5.7±0.7 | 15.3±1.9 |
| Liver | 5.7 ±1.1 | 19.4 ±1.4 | 6.1±0.8 | 50.9±5.7 |
| Kidney | 15.8 ±1.6 | 6.2 ±0.8 | 17.4±1.8 | 15.2±1.3 |
| Spleen | 5.8 ±1.6 | 7.5 ±1.6 | 6.4±0.6 | 18.5±3.1 |
| Lung | 4.2 ±1.0 | 5.0 ±0.4 | 4.3±0.8 | 13.3±1.8 |
| Skin of ear | 2.2 ±0.0 | 1.9 ±0.6 | 2.4±0.1 | 3.2±1.8 |
| Bone + marrow | 3.0 ±0.3 | 1.8 ±0.3 | 3.2±0.2 | 4.5±0.5 |
| Muscle (opp. thigh) | 0.90±0.05 | 0.40±0.05 | | |

Values are mean ± s.d. Animals were killed 18 h after administration of the $^{111}$In labelled agents.

Figure 4), differ from the *in vivo* ROI analyses in which the ratio of $^{111}In$ measured by imaging over the tumour area to that over the contralateral thigh was the same for either administered agent (Figure 3).

The difference between the results of *in vivo* ROI imaging and those obtained by direct counting of dissected tumour and muscle could be due to collection of $^{111}In$ transferrin in the exudate and edematous tissue surrounding the tumour, since this would be detected by *in vivo* scintiscanning but not by direct counting of dissected tumour. In Table III, the fraction of injected $^{111}In$ chloride detected in the tumour area by both methods is compared to the uptake of radiolabelled DHE. It is seen that for $^{111}In$ DHE, tumour uptake as measured by *in vivo* scanning compared well with the counting of dissected tumour tissue. In contrast, a large fraction of the injected $^{111}In$ chloride (about 73%) detected by *in vivo* imaging over the tumour area was not found in the dissected tumour, and therefore must have localised in adjoining tissues. In support of this hypothesis preliminary analysis of fluid exudate in tumours showed $^{111}In$ chloride to concentrate after 18 h to three times that present in the blood.

### *Estimation of dissociation of $^{111}In$-DHE* in vivo

About 80% of the radioactivity extracted from tumours of mice given $^{111}In$ DHE a day earlier moved in TLC at Rf≈0.8, whereas the remaining radioactivity did not migrate in the solvent used. In contrast, all the tumour radioactivity extracted a day after given $^{111}In$ chloride remained at the origin of the TLC strip. This indicated that $^{111}In$ given as the chloride did not bind or remain bound to any tissue constituent that moved in the silica gel TLC with the solvent solution used.

A further estimate of the degree of dissociation was made, based on the high solubility of $^{111}In$ DHE in ethylacetate, its low solubility in the aqueous phase, and the opposite solubility of $^{111}In$ chloride (Table I). In three separate experiments after $^{111}In$ DHE injection, 81.8±2.5% (two mice), 74.0 ± 1.5% (three mice) and 87.9±4.6% (five mice) of the tumour-extractable radioindium appeared in the ethyl acetate phase one day after administration. After a 4-day interval, 74.0 ± 1.5% (three mice) was present in the ethylacetate phase. When $^{111}In$ was given as the chloride, all tumour or liver $^{111}In$ passed into the aqueous phase, and the amount extracted into ethylacetate was insignificant.

Extraction from the tissues was never complete. About 1/2 to 3/4 of tumour $^{111}In$ could be extracted by the technique described from tumours of mice that had received $^{111}In$ DHE. Less was extractable from the liver. For example, in one experiment with five mice, 33.6 ± 4.3% of tumour $^{111}In$ was not extractable, and 74.2 ± 2.4% was not extracted from the liver.

### *Effect of unlabelled DHE on the tissue uptake of $^{111}In$ DHE*

If $^{111}In$ DHE were to be used as a 'tracer' for DHE *in vivo*, it would be important to know the degree to which unlabelled DHE in molecular excess might affect the tissue distribution or the tumour uptake of the radiolabelled DHE. To examine this question, $^{111}In$ DHE and unlabelled DHE were injected simultaneously into tumour bearing mice and the tissue distribution was compared with another group of mice given only $^{111}In$ DHE. The amount of radiolabelled DHE given to each animal was 4 μg, and the total amount of additional unlabelled DHE was 125 μg per mouse.

The results of the experiment are shown graphically in Figure 5. Four animals received $^{111}In$ DHE alone, and another four were given both the radiolabelled and 'cold' DHE simultaneously. The animals were killed after 18 h, and the radioindium content of the tumour and tissues was measured by gamma counting of the dissected tissue samples. When the unlabelled DHE was also given, the mean uptake of $^{111}In$ DHE by the liver was lower by 26% ($P<0.01$) and the tumour content was found to be 42% higher ($P<0.025$), than the uptake of radiolabelled DHE given alone.

**Table III** % tumour uptake of radioindium based on *in vivo* imaging compared with direct counting of dissected tumour

| Agent | *In vivo ROI over tumour area (no. of mice)*[a] | *% uptake per gram of dissected tumour (no. of mice)* |
|---|---|---|
| $^{111}InCl_3$ | 21.3±4.3% (3) | 5.8±0.4% (6) |
| $^{111}In$-DHE | 5.3±1.9% (3) | 5.2±0.7% (7) |

Values are means±s.d. [a]Region of interest (ROI) calculations were made 16 h after injection of the radiolabelled agent using six mice and were calculated from the total counts measured over the tumour divided by the total body count obtained from the scintiscan. [b]The values for dissected tumour (see also Table II) were expressed as % of injected dose per wet weight. Since the total wet mass of the tumour was of the order of 1 g the estimated % total tumour uptake is roughly the same numerically as in this column for comparison with the ROI data.

### Discussion

The use of porphyrins in cancer diagnosis and therapy is based both on their phototoxic properties (Raab, 1900; Haussman, 1911) and on the localisation of fluorescent porphyrins in neoplastic tissues (Policard, 1924; Auler & Banzer, 1942; Figge *et al.*, 1948). A derivative of haematoporphyrin (HPD) has been widely used for tumour detection and photodynamic therapy (PDT) over the past two decades (Lipson *et al.*, 1961; Diamond *et al.*, 1972). However, it has been difficult to identify the specific tumour localising and phototoxic principles of HPD, which is known to be a complex mixture of different prophyrins. Putative candidates are the dihaematoporphyrin ethers or esters (Dougherty *et al.*, 1984; Kessel & Cheng, 1985). A purification product of HPD, known commercially as Photofrin II or DHE, contains less than 20% of inactive monomers and more than 80% of the active porphyrin dimers and oligomers. Use of the term DHE in this report refers to the latter product.

Photodynamic therapy with DHE requires its presence in the tumour. Although fluorescence of tumours has been a hallmark of HPD uptake, it does not necessarily follow that the phototoxic compound taken up into the tumour is identical to the fluorescent agent. Therefore, a direct method of assessing the amount of DHE taken up into the tumour *in vivo* would be of value to determine the probable benefit of PDT, since if the tumour did not taken up the agent, PDT would be ineffective. A radiolabelled DHE that would localise at the tumour site and could be imaged by the gamma-camera would thus be useful for the quantitative estimation of DHE uptake.

A number of workers have investigated the possible use of

**Figure 5** Effect of excess non-labelled DHE on tissue uptake of $^{111}In$-DHE 18 h after i.v. injection via the tail vein of eight tumour-bearing mice. Histogram with light bars represents the tissue uptake of $^{111}In$-DHE. The dark bars present results obtained after giving $^{111}In$-DHE supplemented by excess DHE (molar ratio increased by a factor of 16) at the phototherapeutic level of 1.5 mg DHE $kg^{-1}$.

radiolabelled porphyrins and metalloporphyrins for tumour detection *in vivo* (Figge *et al.*, 1948; Manganiello & Figge, 1951; Bases *et al.*, 1958; Winkelman *et al.*, 1962), but with varying degrees of success. A major problem with the use of these agents has been their high degree of accumulation in the liver, spleen and kidneys (Hambright *et al.*, 1975; Denechaud *et al.*, 1981; Zanelli & Kaelin, 1981). Attempts to image tumours with $^{111}$In labelled porphyrins have been limited (Vaum *et al.*, 1982; Foster *et al.*, 1985). In summary of past studies, encouraging tumour localisations of radiolabelled porphyrins have been demonstrated in animal models, but the limited human studies have not shown tumour localisation, possibly due to dissociation of the radiolabel from the porphyrin *in vivo* or to a metallation-induced loss of the tumour localising property of the porphyrin preparation.

In the present experiments using DBA/2J mice bearing transplanted rhabdomyosarcomas, marked tumour uptake of $^{111}$In DHE was observed. The DHE was radiolabelled with $^{111}$In by a method adapted from that of Lavallee and Fawwaz (1986) in which heating of DHE was kept not greater than 65°C. The following four experimental findings suggest that $^{111}$In DHE has biological properties similar to those of DHE: (a) The tissue and tumour distribution $^{111}$In DHE *in vivo*, as measured from the radioindium content of perfused and dissected tissues, was very similar to that found for $^{14}$C and $^{3}$H-HPD (Gomer & Dougherty, 1979) and $^{14}$C-DHE (Bellnier *et al.*, 1988) as shown in Figure 4. (b) About 80% of the tumour-extractable $^{111}$In that had been administered bound to DHE remained after 1 day or more associated with the porphyrin *in vivo*, as estimated by its extraction with ethylacetate, whereas $^{111}$In given as the chloride was not extracted into the ethylacetate phase. (c) Tissue distribution studies of $^{111}$In DHE were carried out using $^{111}$In chloride as control to rule out the possibility that $^{111}$In may dissociate *in vivo* from DHE with resultant $^{111}$In uptake by the tumour (Saha & Farrer, 1975; Ando *et al.*, 1982). The tissue distribution of the two agents differed markedly: the former localised primarily in the liver with lower uptake by other tissues; in contrast, the latter rapily accumulated in the kidneys, other tissues showing greater uptake and slower clearance. This is further evidence that the two $^{111}$In preparations are handled differently *in vivo*. (d) The presence of excess unlabelled DHE did not prevent the tumour uptake of $^{111}$In DHE. Indeed some enhancement of tumour uptake was observed, with reduced uptake of $^{111}$In DHE into the liver (Figure 5).

The mechanism of the cold DHE effect is not clear. It might be due to the saturation of binding sites in the liver whose affinity for cold DHE is greater than for the $^{111}$In labelled DHE, thus causing increased uptake of the latter by the tumour. Perhaps this is a clue to structural modifications that could be made to DHE to reduce liver binding and enhance tumour uptake.

These findings suggest that $^{111}$In DHE may be useful as a quantitative indicator of tumour DHE uptake. It would be premature to use the term 'tracer', which implies a very close correspondence between the biological properties of the agents; this may not be the case for $^{111}$In DHE. The experiment using cold DHE is excess showing uptake of $^{111}$In DHE which was significantly increased over the tumour but reduced over the liver, thus suggestive of interference by cold DHE in the biological uptake of the radiolabelled porphyrin. Therefore, the term 'tracer' in this case must be used with caution.

Following administration of $^{111}$In chloride, measurements of $^{111}$In over the tumour area by region of interest (ROI) *in vivo* scanning differed from the results obtained by direct counting of perfused and dissected tissues. Of the total $^{111}$In 21.3% was detected over the tumour area by gamma-camera imaging, but only 5.8% $g^{-1}$ was measured in the dissected tumour tissue (Table III). The remaining tracer must have been retained in the oedematous tissue and exudate surrounding the tumour, as shown by preliminary measurements of tumour exudate in $^{111}$In chloride treated mice showing three times the blood concentration of $^{111}$In (unpublished results). In contrast, $^{111}$In DHE detected over the tumour area by gamma-camera imaging corresponded well to the direct counting of the dissected tumour tissue, suggesting the possibility that gamma-camera imaging can be used to estimate the tumour uptake of radiolabelled DHE. The comparative cellular and extracellular tumour distribution of $^{111}$In DHE and $^{111}$In given as the chloride was not addressed in this study and clearly requires further investigation.

The much slower tissue turnover of $^{111}$In administered as the chloride (Figure 2) is presumably due to its binding to transferrin in the plasma, with long lived vascular and extravascular components (Hosain *et al.*, 1969; McIntyre *et al.*, 1974). In contrast, the rapid turnover of $^{111}$In DHE corresponds to the fast blood clearance of $^{14}$C-DHE observed by Bellnier *et al.* (1988) and is likely to be due to rapid passage of the agent into the gut without an hour after administration. In support of this, we have demonstrated (unpublished) marked passage of radioindium into rabbit gut within an hour of $^{111}$In DHE i.v. injection.

Metallation of hematoporphyrin in the tetrapyrrole ring is considered to have an inhibitory effect on tumour affinity for the agent (Zanelli & Kaelin, 1981; Wang *et al.*, 1981). We speculate that the observed tumour uptake is due in part to incomplete $^{111}$In labelling of DHE, which contains more than 80% dimers and oligomers, and that the unmetallated porphyrins present within the radiolabelled dimers or oligomers may retain tumour affinity. In support of this hypothesis, recent preliminary experiments (unpublished) have shown that when $^{111}$In DHE was additionally labelled with stable indium, tissue and tumour uptake of the radiolabelled agent was much reduced.

Significantly elevated tissue:muscle ratios ($P<0.005$) for $^{111}$In DHE in comparison to $^{111}$In chloride were found for tumour, lung, spleen and liver, though not for skin (ear), bone (marrow) or kidney (Table II). The relatively increased uptake by the liver, spleen, lungs and tumour indicates the existence of concentrative mechanisms. That the former three organs are involved suggests uptake of radiolabelled DHE by the reticuloendothelial system (Bugelski *et al.*, 1981), but another uptake mechanism by tumour may also be involved. Kessel (1986) has described the binding of tumour-localising porphyrins to high and low density lipoproteins (HDL and LDL) which have been shown to transport HPD (Jori *et al.*, 1984; Reyftmann *et al.*, 1984). Increased LDL receptors have been associated with neoplastic cells (Norata *et al.*, 1984; Gal *et al.*, 1981). A combination of altered vascular permeability, poor lymphatic drainage and a lipoprotein receptor mechanism could lead to increased tumour cell uptake.

The question obviously arises as to whether $^{111}$In DHE might be useful as a tumour imaging agent. We do not have enough information to answer this, except that its relatively rapid clearance from the uninvolved tissues might improve the resolution of those tumours which take up the agent. The heavy uptake by liver and spleen would, however, be a drawback. More studies with tumours of human origin in addition to animals models are clearly necessary.

The main value of the present observations appears to lie in the possibility of estimating *in vivo* the degree of porphyrin uptake by tumours in phototherapy for cancer. Needless to say, mice are not men, and human tumour biology differs from that of transplanted murine tumours. In view of the lack of past success using various radiolabelled porphyrins in the limited patient studies that have so far been carried out, caution is advisable in making any predictions as to how these agents may behave in humans.

We express our thanks to Dr D. Lavallee for graciously helping in the initial preparations of the $^{111}$In DHE. We are grateful to Dr H. Dougan and Dr D. Lyster for their suggestions and the use of their facilities. Dr W. Ammann and Dr B. Lentle made gamma-camera facilities available to the study. Dr J. Chow and Dr D. Liu helped us to solve some of the chemical problems involved in the preparation of the radiolabelled DHE. Dr G.D. Zanelli's suggestions regarding the significance of partial metallation were appreciated. Support for M.R.Q. from the UICC for an Eleanor Roosevelt Cancer Fellowship is gratefully acknowledged.

### References

ANDO, A., ANDO, I., HIRAKI, T., TAKESHITA, M. & HISADA, K. (1982). Mechanism of tumor and liver concentration of In-111 and Yb-169: In-111 and Yb-169 binding substances in tumor tissues and liver. *Eur. J. Nucl. Med.*, **7**, 298.

AULER, H. & BANZER, G. (1942). Untersuchungen uber die Rolle der Porphyrine bei geschwulstkranken Menschen und Tieren. *Z. Krebsforsch.*, **53**, 65.

BASES, R., BRODIE, S.S. & RUBENFELDS, S. (1958). Attempts at tumor localization using Cu-64 labeled copper porphyrins. *Cancer*, **11**, 259.

BELLNIER, D.A., HO, Y-K., PANDEY, R.K., MISSERT, J.R. & DOUGHERTY, T.J. (1988). Distribution and elimination of Photofrin II in mice. *Photochem. Photobiol.*, **50**, 221.

BUGELSKI, P.J., PORTER, C.W. & DOUGHERTY, T.J. (1981). Autoradiographic distribution of hematoporphyrin derivatives in normal and tumor tissue of the mouse. *Cancer Res.*, **41**, 4606.

DENECHAUD, M., LAVAL, M., DABADIE, M., DUCASSOU, D. & POMMIER, J.C. (1981). Interet d'une metallo-porphyrine en cancerologie experimentale. *Bull. Cancer (Paris)*, **68**, 40.

DIAMOND, I., GRANELLI, S.G., MCDONAGH, A.F., NIELSEN, S., WILSON, C.B. & JAENICKE, R. (1972). Photodynamic therapy of malignant tumours. *Lancet*, **ii**, 1175.

DOUGHERTY, T.J. (1987). Photosensitizers: therapy and detection of malignant tumors. Yearly review. *Photochem. Photobiol.*, **45**, 879.

DOUGHERTY, T.J. & MANG, T.S. (1987). Characterization of intratumoral porphyrin following injection of hematoporphyrin derivative or its purified component. *Photochem. Photobiol.*, **46**, 67.

DOUGHERTY, T.J., POTTER, W.R. & WEISHAUPT, K.R. (1984). The structure of the active component of hematoporphyrin derivative. In *Porphyrin Localization and Treatment of Tumors*, Doiron, D.R. & Gomer, C.J. (eds) p. 301. Alan R. Liss: New York.

FIGGE, F.H.J., WELAND, G.S. & MANGANIELLO, L.O.J. (1948). Cancer detection and therapy. Affinity of neoplastic, embryonic and traumatized tissues for porphyrins and metalloporphyrins. *Proc. Soc. Exp. Biol. Med.*, **68**, 640.

FOSTER, N., WOO, D.V., KALTOVICH, F., EMRICH, J. & LJUNGQUIST, C. (1985). Delineation of a transplanted malignant melanoma with In-111 labeled porphyrin. *J. Nucl. Med.*, **26**, 756.

GAL, D., MCDONALD, P.C., PORTER, J.C. & SIMPSON, E.R. (1981). Cholesterol metabolism in cancer cells in monolayer culture: III Low density lipoprotein metabolism. *Int. J. Cancer*, **29**, 315.

GOMER, C.J. & DOUGHERTY, T.J. (1979). Determination of H-3- and C-14-hematoporphyrin derivative distribution in malignant and normal tissue. *Cancer Res.*, **39**, 146.

HAMBRIGHT, P., FAWWAZ, R., VALK, P., MCRAE, J. & BEARDEN, A.J. (1975). The distribution of various soluble radioactive metalloporphyrins in tumour bearing rats. *Bioinorg. Chem.*, **5**, 87.

HAUSSMAN, W. (1911). Die sensibilirende Wirkung des Hämatoporphyrins. *Biochem. Z.*, **30**, 276.

HOSAIN, F., MCINTYRE, P.A., POULOSE, K., STERN, H.S. & WAGNER, H.N. Jr (1969). Binding of trace amounts of ionic indium-113m to plasma transferrin. *Clin. Chim. Acta*, **24**, 69.

JORI, G., BELTRAMINI, M., REDDI, E. & 5 others (1984). Evidence for a major role of lipoproteins as hematoporphyrin carriers *in vivo*. *Cancer Lett.*, **24**, 291.

KESSEL, D. (1986*a*). Proposed structure of the tumor localizing fraction of HPD (hematoporphyrin derivative). *Photochem. Photobiol.*, **44**, 193.

KESSEL, D. (1986*b*). Porphyrin–lipoprotein association as a factor in porphyrin localization. *Cancer Lett.*, **33**, 183.

KESSEL, D. & CHENG, M.-L. (1985). On the preparation and properties of dihematoporphyrin ether, the tumor localizing component of HPD. *Photochem. Photobiol.*, **41**, 277.

LAVALLEE, D.K. & FAWWAZ, R. (1986). The synthesis and characterization of In-111 hematoporphyrin derivative. *Nucl. Med. Biol. (Int. J. Appl. Instr. B)*, **13**, 639.

LIPSON, R.L., BALDES, E.J. & OLSEN, A.M. (1961). The use of a derivative of hematoporphyrin in tumor detection. *J. Natl Cancer Inst.*, **26**, 1.

MANGANIELLO, L.O.J. & FIGGE, F.H.J. (1951). Cancer detection and therapy. II Methods of preparation and biological effects of metallo-porphyrin. *Bull. Sch. Med. Univ. Maryland*, **36**, 3.

MCINTYRE, P.A., LARSON, S.M., EIKMAN, E.A., COLMAN, M., SCHEFFEL, U. & HODKINSON, B.A. (1974). Comparison of metabolism of iron-transferrin and indium-transferrin by the erythropoietic marrow. *J. Nucl. Med.*, **15**, 856.

NORATA, G., CANTI, G., RICCI, L., NICOLIN, A., TREZZI, E. & CASTAPANO, A.L. (1984). *In vivo* assimilation of low density lipoproteins by a fibrosarcoma tumour line in mice. *Cancer Lett.*, **25**, 203.

POLICARD, A. (1924). Etude sur les aspects offerts par des tumeurs experimentales examinees a la lumiere de Wood. *C. R. Soc. Biol.*, **91**, 1423.

RAAB, O. (1900). Ueber die Wirkung fluorescirender Stoffe auf Infusorien. *Z. Biol.*, **39**, 524.

REYFTMANN, J., MORLIERE, P., GOLDSTEIN, S., SANTUS, R., DUBERET, L. & LAGRANGE, D. (1984). Interactions of human serum low density lipoproteins with porphyrins: a spectroscopic and photochemical study. *Photochem. Photobiol.*, **40**, 721.

SAHA, G.B. & FARRER, P.A. (1975). A comparative study of tumor uptake and tissue distribution of Ga-67 citrate, In-111 $Cl_3$ and Hg-197 $Cl_2$. In *Radiopharmaceuticals*, Subramanian, G., Rhodes, B.A., Cooper, J.F. & Sodd, V.J. (eds) p. 435. Soc. Nuc. Med: New York.

VAUM, R., HEINDEL, N.D., BURNS, H.D., EMRICH, J. & FOSTER, N. (1982). Synthesis and evaluation of an In-111 labeled porphyrin for lymph node imaging. *J. Pharmaceut. Sci.*, **71**, 1223.

WANG, T.S.T., FAWWAZ, R.A. & TOMASHEFSKY, P. (1981). Metalloporphyrin derivatives: Structure-localization properties. In *Radiopharmaceuticals: structure, localization properties*, Spencer, R.P. (ed.) p. 225. Grune and Stratton: New York.

WINKELMAN, J., MCAFEE, J.G., WAGNER, H.N. & LONG, R.G. (1962). The synthesis of Co-57 tetraphenylporphinesulfonate and its use in the scintillation scanning of neoplasms. *J. Nucl. Med.*, **3**, 249.

ZANELLI, G.D. & KAELIN, A.C. (1981). Synthetic porphyrins as tumour localizing agents. *Br. J. Radiol.*, **54**, 403.