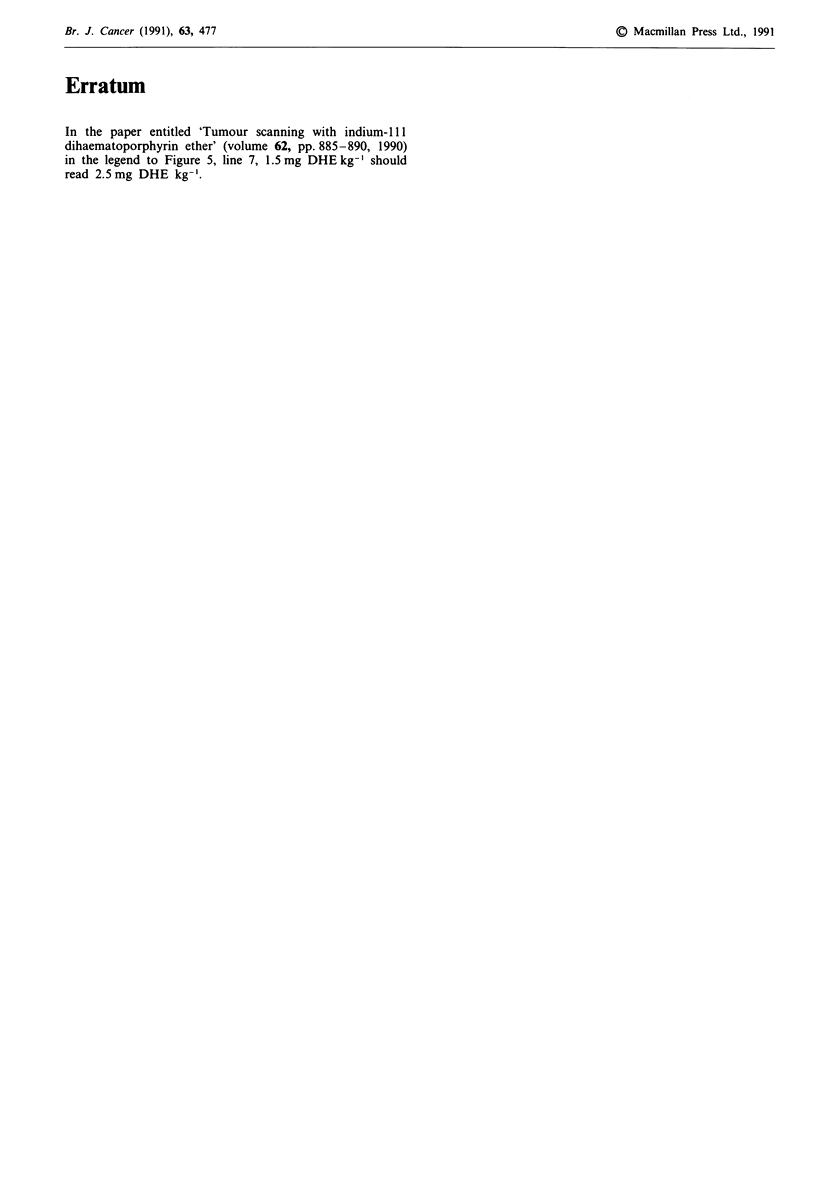# Erratum

**Published:** 1991-03

**Authors:** 


*Br. J. Cancer* (1991), **63**, 477 © Macmillan Press Ltd., 1991

### Erratum

In the paper entitled 'Tumour scanning with indium-111 dihaematoporphyrin ether' (volume **62**, pp. 885–890, 1990) in the legend to Figure 5, line 7, 1.5 mg DHE $kg^{-1}$ should read 2.5 mg DHE $kg^{-1}$.